# Periostin and rheumatic diseases: early insights from a systematic review and meta-analysis

**DOI:** 10.1007/s10238-025-01615-0

**Published:** 2025-03-07

**Authors:** Arduino A. Mangoni, Angelo Zinellu

**Affiliations:** 1 Discipline of Clinical Pharmacology, College of Medicine and Public Health, Flinders University, Adelaide, Australia; 2 Department of Clinical Pharmacology, Flinders Medical Centre, Southern Adelaide Local Health Network, Adelaide, Australia; 3 Department of Biomedical Sciences, University of Sassari, Sassari, Italy

**Keywords:** Angiogenesis, Autoimmunity, Biomarkers, Fibrosis, Inflammation, Periostin, Rheumatic diseases

## Abstract

Periostin regulates angiogenesis, inflammation, and fibrosis, key processes in the pathophysiology of rheumatic diseases (RDs). However, its association with RDs has not been assessed. We conducted a systematic review and meta-analysis of studies reporting circulating periostin in RD patients and healthy controls. We searched electronic databases from inception to 30 November 2024 for relevant articles and assessed the risk of bias and the certainty of evidence using the JBI critical appraisal checklist and GRADE, respectively. In 12 eligible studies, there was a non-significant trend towards higher periostin concentrations in RD patients (standard mean difference, SMD = 0.46, 95% CI −0.07 to 0.98, *p* = 0.089; *I*^2^ = 94.2%, *p* < 0.001). The results were stable in sensitivity analysis. There were no significant associations between the SMD and age, male-to-female ratio, number of participants, or publication year. However, we observed significant periostin elevations in studies investigating systemic sclerosis and rheumatoid arthritis but not osteoarthritis. Significant periostin reductions were observed in studies investigating ankylosing spondylitis and dermatomyositis. Furthermore, the SMD was significant in studies conducted in America, but not Asia or Europe. Our study suggests significant periostin elevations in rheumatoid arthritis and systemic sclerosis. Such elevations may reflect a more pronounced dysregulation of angiogenesis and fibrosis when compared to other RDs. Further research is warranted to investigate periostin concentrations in a wide range of RDs with various inflammatory, angiogenic, and fibrotic features and whether periostin is useful for diagnosis, prognosis, and monitoring in this patient group (PROSPERO registration number: CRD42024623501).

**Supplementary Information:**

The online version contains supplementary material available at 10.1007/s10238-025-01615-0.

## Introduction

Rheumatic diseases (RDs) are a group of chronic and disabling conditions affecting multiple organs and systems that are characterized by a predominantly autoimmune (e.g. rheumatoid arthritis and systemic lupus erythematosus), mixed autoimmune-autoinflammatory (e.g. ankylosing spondylitis and psoriatic arthritis), or autoinflammatory (e.g. familial Mediterranean fever) process [1–4]. Regardless of the relative importance of autoinflammation vs. autoimmunity, a dysregulation of inflammatory pathways is the mainstay of RDs [5]. Such dysregulation has led to the routine use of inflammatory biomarkers, e.g. C-reactive protein and erythrocyte sedimentation rate, for diagnosis and monitoring [6–9]. However, limitations in their diagnostic accuracy have prompted the search for alternative biomarkers [6, 10–12].

Experimental and clinical studies have convincingly shown that several types of RDs are characterized, in addition to excess inflammation, by dysregulated angiogenesis and fibrosis. Significant elevations in the circulating concentrations of the vascular endothelial growth factor (VEGF), a key mediator of angiogenesis, have been observed in several types of RDs, e.g. rheumatoid arthritis, osteoarthritis, ankylosing spondylitis, psoriatic arthritis, systemic lupus erythematosus, systemic sclerosis, and Sjögren’s syndrome [13–19]. Dysregulated VEGF concentrations favour synoviocyte apoptosis [20], osteoclast activation [21], and macrophage stimulation [22, 23]. Excess fibrosis also represents a key feature in many RDs and is considered the result of the overproduction of growth factors, proteolytic enzymes, angiogenic factors, and pro-fibrotic cytokines [24]. Notably, excess fibrosis often leads to significant tissue remodelling and organ dysfunction which, in turn, increase morbidity and mortality in several types of RDs, particularly rheumatoid arthritis and systemic sclerosis [25, 26]. Therefore, the identification of biomarkers reflecting the presence of angiogenic and fibrotic alterations, in addition to excess inflammation, might be particularly useful in diagnosing and monitoring RDs.

Periostin, a 90 kDa matricellular protein first discovered in 1993 under the name of osteoblast-specific-factor-2 [27], regulates embryonic formation, the structure of the extracellular matrix, bone and teeth homeostasis, and the structural and functional properties of collagen-rich connective tissues such as the heart valves and tendons [28, 29]. In vitro and in vivo studies have also reported a significant upregulation of periostin in pathological states characterized by inflammation, fibrosis, and dysregulated angiogenesis, e.g. allergy, asthma, and cancer [30–34]. Periostin can be measured in plasma and serum using enzyme-linked immunosorbent assays (ELISA). Its concentrations have been shown to predict clinical outcomes in cancer, renal disease, and idiopathic pulmonary fibrosis, suggesting its possible use as a biomarker in these and other conditions, e.g. allergic diseases [30, 35–39].

Given the increasing interest in the pathophysiological role of periostin as well as the lack of a comprehensive appraisal of the available evidence regarding its association with RDs, we conducted a systematic review and meta-analysis of studies investigating circulating periostin in patients with RDs and healthy controls. We hypothesized that the presence of RDs was associated with a significant increase in periostin concentrations, reflecting a state of dysregulated inflammation, angiogenesis and fibrosis. We also investigated associations between the effect size of the between-group differences in periostin concentrations and several demographic and clinical characteristics, including specific types of RDs.

## Materials and methods

### Search strategy and study selection

We systematically searched the electronic databases, PubMed, Web of Science, and Scopus, from their inception to 30 November 2024, for relevant articles according to the following terms (see Supplementary Table 1 for the search strategies used in individual databases): “periostin” OR “POSTN” OR “osteoblast-specific factor 2″ OR “OSF-2″ AND “rheumatic diseases” OR “rheumatoid arthritis” OR “psoriatic arthritis” OR “reactive arthritis” OR “ankylosing spondylitis” OR “systemic lupus erythematosus” OR “systemic sclerosis” OR “scleroderma” OR “Sjögren’s syndrome” OR “connective tissue diseases” OR “vasculitis” OR “Bechet’s disease” OR “idiopathic inflammatory myositis” OR “polymyositis” OR “dermatomyositis” OR “gout” OR “pseudogout” OR”systemic vasculitis” OR “ANCA-associated vasculitis” OR “Takayasu arteritis” OR “polyarteritis nodosa” OR “osteoarthritis” OR “fibromyalgia” OR “granulomatous polyangiitis” OR”Henoch-Schonlein purpura” OR “Wegener’s granulomatosis” OR “familial Mediterranean fever” OR “polymyalgia rheumatica”.

Two investigators independently screened the abstracts to determine relevance. If a publication was considered potentially relevant, the full text of the article was independently reviewed. A third investigator was involved in case of disagreement. The inclusion criteria were: (i) the investigation of circulating periostin concentrations in patients with RD diagnosed according to accepted guidelines and healthy controls in case–control studies, (ii) the recruitment of adult participants, and (iii) the availability of the full text of the publication in English language. The exclusion criteria were: (i) review articles or research letters that did not report original research data, (ii) cellular or animal studies, (iii) studies including participants under 18 years (as there is evidence that periostin concentrations are strongly correlated with age at a young age, potentially introducing significant bias) [40, 41], and (iv) studies lacking a control group. The references of individual articles were hand searched for additional studies.

The following data were independently extracted from each article two investigators and transferred into separate spreadsheets for comparison and analysis: year of publication, details of the first author, country where the study was conducted, sample size, age, male-to-female ratio, and type of RD. We assessed the risk of bias using the Joanna Briggs Institute (JBI) critical appraisal checklist for analytical cross-sectional studies [42]. Studies addressing ≥ 75%, ≥ 50% and < 75%, and < 50% of checklist items were considered having low, moderate, or high risk of bias, respectively. We evaluated the level of the certainty of evidence according to the Grades of Recommendation, Assessment, Development, and Evaluation (GRADE) Working Group system [43]. This tool considers the study design, risk of bias, presence of unexplained heterogeneity, indirectness of evidence, imprecision of results, effect size (small, standard mean difference, SMD < 0.5, moderate, SMD 0.5–0.8, and large, SMD > 0.8) [44], and probability of publication bias. We followed the Preferred Reporting Items for Systematic Reviews and Meta-Analyses (PRISMA) 2020 statement (Supplementary Table 1) [45]. We registered the study protocol in the International Prospective Register of Systematic Reviews (PROSPERO registration number: CRD42024623501).

### Statistical analysis

We calculated standardized mean differences (SMDs) and 95% confidence intervals (CIs) to generate forest plots comparing periostin concentrations in RD patients and controls. A p-value of < 0.05 was considered statistically significant. If required, means and standard deviations were extrapolated from medians and interquartile or full ranges, as previously reported [46]. The heterogeneity of SMD across studies was evaluated using the Q statistic (the significance level was set at *p* < 0.10). Heterogeneity was classified as low when *I*^2^ ≤ 25%, moderate when 25% < *I*^2^ < 75%, and high when *I*^2^ ≥ 75% [47]. We used a random-effects model based on the inverse-variance method if high heterogeneity was present.

We performed sensitivity analysis to assess the influence of each study on the overall effect size by sequentially excluding individual studies [48]. We assessed the presence of publication bias using Begg’s adjusted rank correlation test and Egger’s regression asymmetry test (significance level set at *p* < 0.05) [49, 50]. We also used the Duval and Tweedie “trim-and-fill” procedure to further examine and potentially correct publication bias [51]. We conducted univariate meta-regression and subgroup analyses to investigate associations between the effect size and year of publication, country or continent where the study was conducted, sample size, age, male-to-female ratio, and type of RDs. Statistical analyses were performed using Stata 14 (Stata Corp., College Station, TX, USA).

## Results

### Study selection and characteristics

**Fig. 1:**
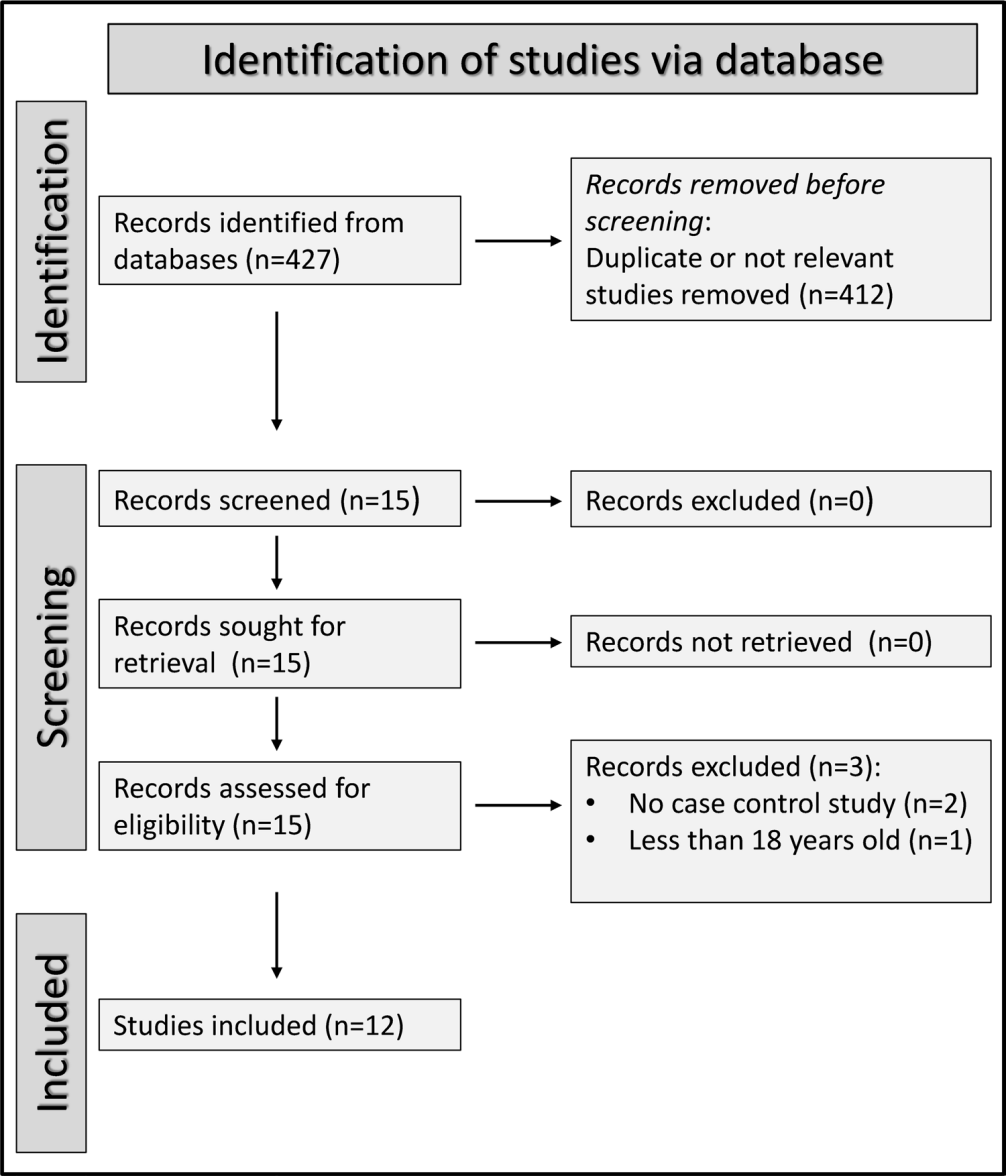
PRISMA 2020 flow diagram of study screening and selection

Figure 1 describes the flowchart of the screening and selection process. After initially identifying 427 studies, we excluded 412 in the preliminary screening phase as they were either duplicates from different electronic databases or reported irrelevant information. Following a comprehensive review of the full text of the remaining 15 articles, a further three were excluded because of the absence of a control group (*n* = 2) or the inclusion of participants under 18 years (*n* = 1). Therefore, 12 studies, published between 2012 and 2024, were selected for analysis [52–63]. There was full concordance between the two investigators involved in the search and data extraction.

As shown in Table 1, there were 13 group comparators including 851 RD patients (mean age 52 years, 82% females) and 638 healthy controls (mean age 45 years, 52% females) [52–63]. Six studies were conducted in Asia [52, 53, 55, 57, 60, 61], four in Europe [54, 56, 58, 63], and two in America [59, 62]. Four group comparators included individuals with systemic sclerosis [52, 59, 62, 63], three with rheumatoid arthritis [56, 61], two with anchylosis spondylitis [54, 55], two with dermatomyositis [57, 58], and two with osteoarthritis [53, 60]. All studies measured periostin using an ELISA in serum, except one which measured plasma [53].

**Table 1:** Characteristics of the studies investigating periostin in patients with rheumatic diseases and healthy controls

Study	Controls				Patients with rheumatic diseases				Disease type
	n	Age (years)	M/F	Periostin (Mean ± SD)	n	Age (years)	M/F	Periostin (Mean ± SD)	
Yamaguchi et al., Japan [52]	66	NR	NR	36 ± 25	56	60.8	7/49	106 ± 39	SSc
Honsawek et al., Thailand [53]	20	68.7	5/15	145.7 ± 29.5	90	70	16/74	149.7 ± 84	OA
Sakellariou et al., Greece [54]	36	39.3	2/34	291.4 ± 50	65	41.3	4/61	234.4 ± 60.4	AS
Solmaz et al., Turkey [55]	48	41	12/36	46.6 ± 50	97	38	21/76	32.4 ± 19.2	AS
Kerschan-Schindl et al., Austria [56]	24	62	0/24	1256 ± 894	24	61	0/24	3208 ± 1453	RA
Chen et al., China [57]	30	47.7	4/26	32.3 ± 13.7	64	47.7	16/48	23.6 ± 13,0	DM
Kerschan-Schindl et al., Austria [58]	20	61.7	3/17	4.4 ± 3.5	20	65.7	2/8	3.7 ± 3.0	DM
El-Adili et al., USA [59]	22	NR	NR	66.97 ± 61.72	106	55.7	18/88	197 ± 162	SSc
Tan et al., China [60]	18	38.3	NR	907.4 ± 153.1	32	68.2	NR	894.2 ± 131.1	OA
Matama et al., Japan [61]	137	40.3	91/46	63.7 ± 17.2	20	66.3	4/16	71.4 ± 23.1	RA
Matama et al., Japan [61]	137	40.3	91/46	63.7 ± 17.2	19	70.3	6/13	105.6 ± 50.5	RA
Sheng et al., USA [62]	50	59	32/18	33.2 ± 7.47	208	48	40/168	59.6 ± 34.4	SSc
Luca et al., Italy [63]	30	NR	NR	27.7 ± 7.3	50	53.1	17/33	32.7 ± 8.0	SSc

AS, ankylosing spondylitis; DM, dermatomyositis; M/F, male-to-female ratio; NR, not reported; OA, osteoarthritis; RA, rheumatoid arthritis; SSc, systemic sclerosis

The risk of bias was low in seven studies [53–56, 58, 60, 61] and moderate in the remaining five [52, 57, 59, 62, 63] (Table 2). The initial level of the certainty of evidence was ranked as low (level 2) because of the cross-sectional design of the selected studies.

**Table 2:** Assessment of the risk of bias using the Joanna Briggs Institute critical appraisal checklist for analytical cross-sectional studies

Study	Were the inclusion criteria clearly defined?	Were the subjects and the setting described in detail?	Was the exposure measured in a reliable way?	Were standard criteria used to assess the condition?	Were confounding factors identified?	Were strategies to deal with confounding factors stated?	Were the outcomes measured in a reliable way?	Was appropriate statistical analysis used?	Risk of bias
Yamaguchi et al. [52]	No	Yes	Yes	Yes	No	No	Yes	Yes	Moderate
Honsawek et al. [53]	Yes	Yes	Yes	Yes	Yes	Yes	Yes	Yes	Low
Sakellariou et al. [54]	Yes	Yes	Yes	Yes	Yes	Yes	Yes	Yes	Low
Solmaz et al. [55]	Yes	Yes	Yes	Yes	Yes	Yes	Yes	Yes	Low
Kerschan-Schindl K et al. [56]	Yes	Yes	Yes	Yes	No	No	Yes	Yes	Low
Chen et al. [57]	No	Yes	Yes	Yes	No	No	Yes	Yes	Moderate
Kerschan-Schindl et al. [58]	Yes	Yes	Yes	Yes	No	No	Yes	Yes	Low
El-Adili et al. [59]	No	Yes	Yes	Yes	No	No	Yes	Yes	Moderate
Tan et al. [60]	Yes	Yes	Yes	Yes	Yes	Yes	Yes	Yes	Low
Matama et al. [61]	Yes	Yes	Yes	Yes	No	No	Yes	Yes	Low
Sheng et al. [62]	No	Yes	Yes	Yes	No	No	Yes	Yes	Moderate
Luca et al. [63]	No	Yes	Yes	Yes	No	No	Yes	Yes	Moderate

### Results of individual studies and syntheses

**Fig. 2:**
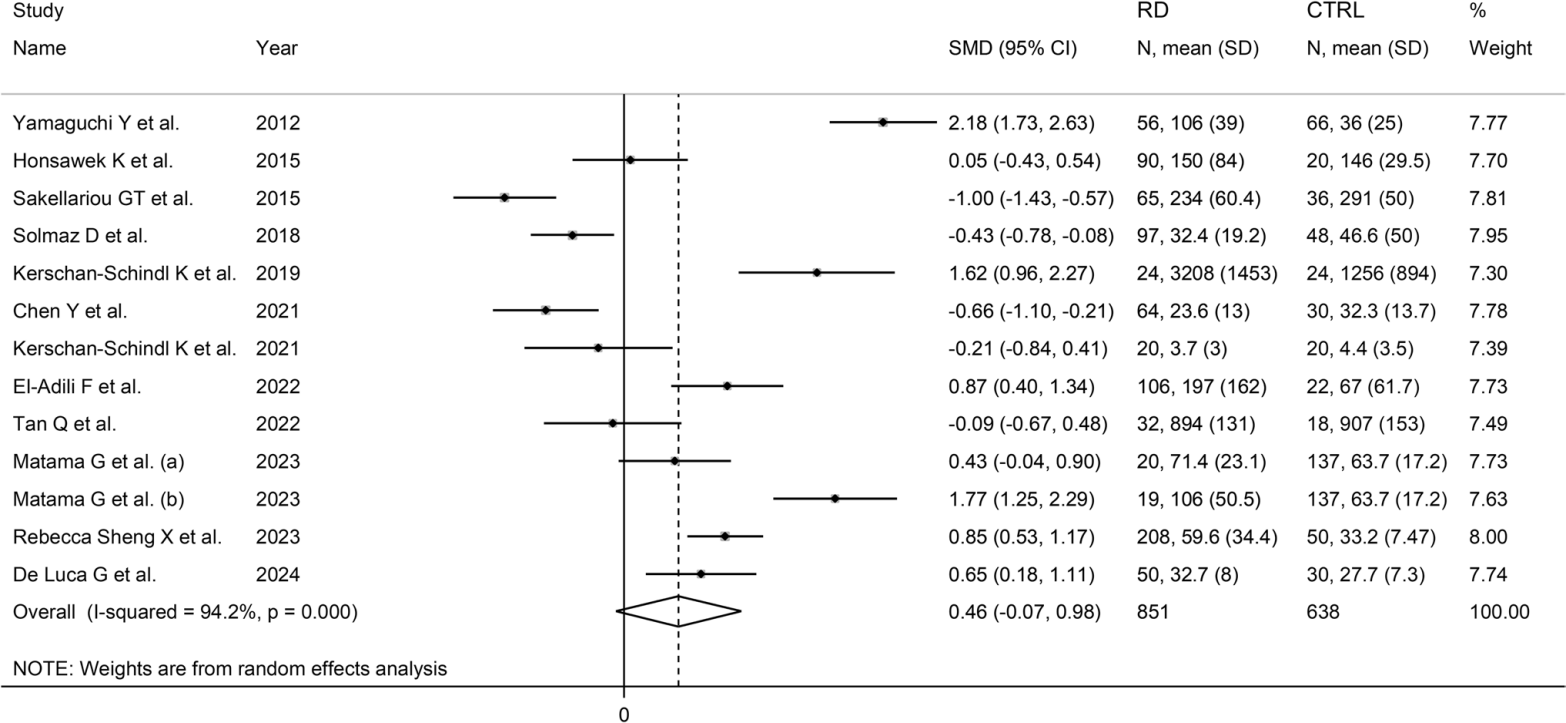
Forest plot of studies investigating periostin in patients with rheumatic diseases and healthy controls

**Fig. 3:**
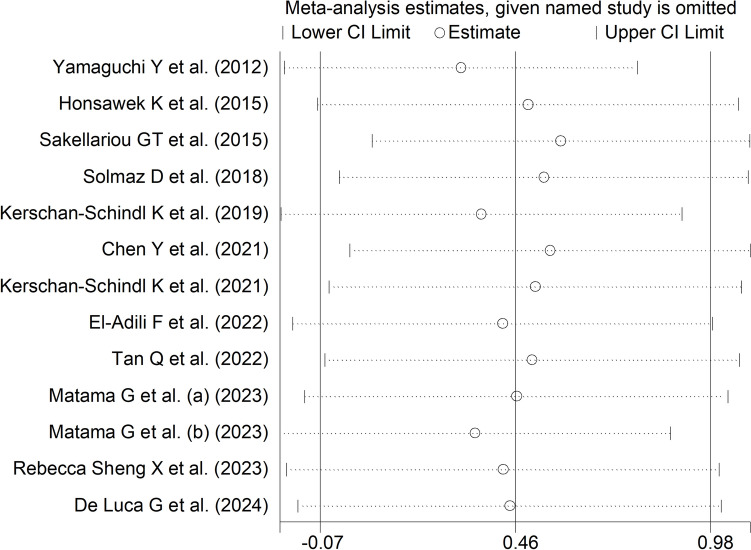
Sensitivity analysis of the association between periostin and rheumatic diseases. For each study, the effect size (hollow circles) corresponds to an overall effect size computed from a meta-analysis excluding that study

The forest plot (Fig. 2) showed a non-significant trend towards higher periostin concentrations in RD patients when compared to controls (SMD = 0.46, 95% CI −0.07 to 0.98, *p* = 0.089; *I*^2^ = 94.2%, *p* < 0.001). Sensitivity analysis (Fig. 3) showed that the corresponding pooled SMD values were not significantly altered when individual studies were sequentially removed, with the effect size ranging between 0.31 and 0.58.

### Publication bias

**Fig. 4:**
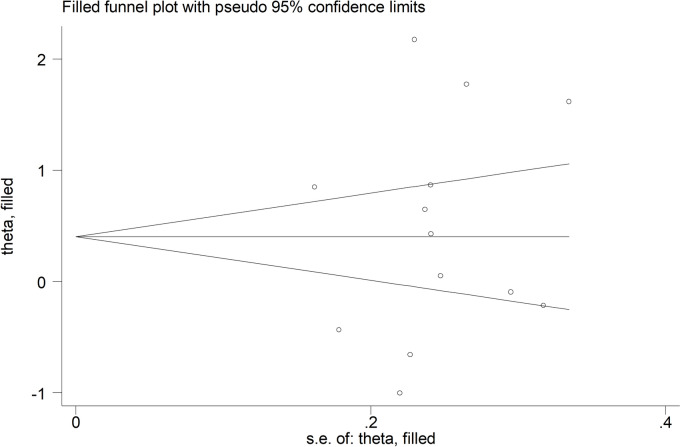
Funnel plot of studies investigating the association between periostin and rheumatic diseases after “trimming-and-filling”. Dummy studies and genuine studies are represented by enclosed circles and free circles, respectively

There was no significant publication bias according to Begg’s (*p* = 0.67) or Egger’s (*p* = 0.49) test. Accordingly, the “trim-and-fill” method did not identify any missing study to be added to the funnel plot to ensure symmetry (Fig. 4).

### Meta-regression and subgroup analysis

**Fig. 5:**
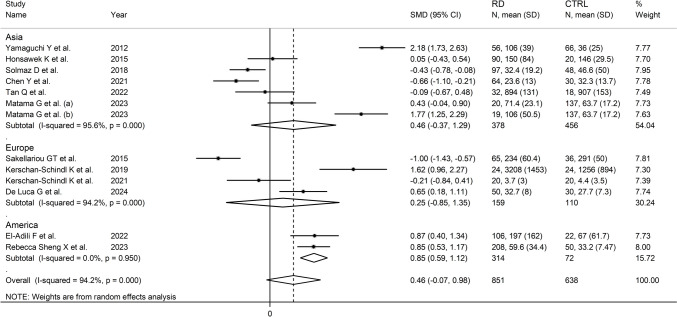
Forest plot of studies investigating periostin in patients with rheumatic diseases and healthy controls according to the geographical area where the study was conducted

**Fig. 6:**
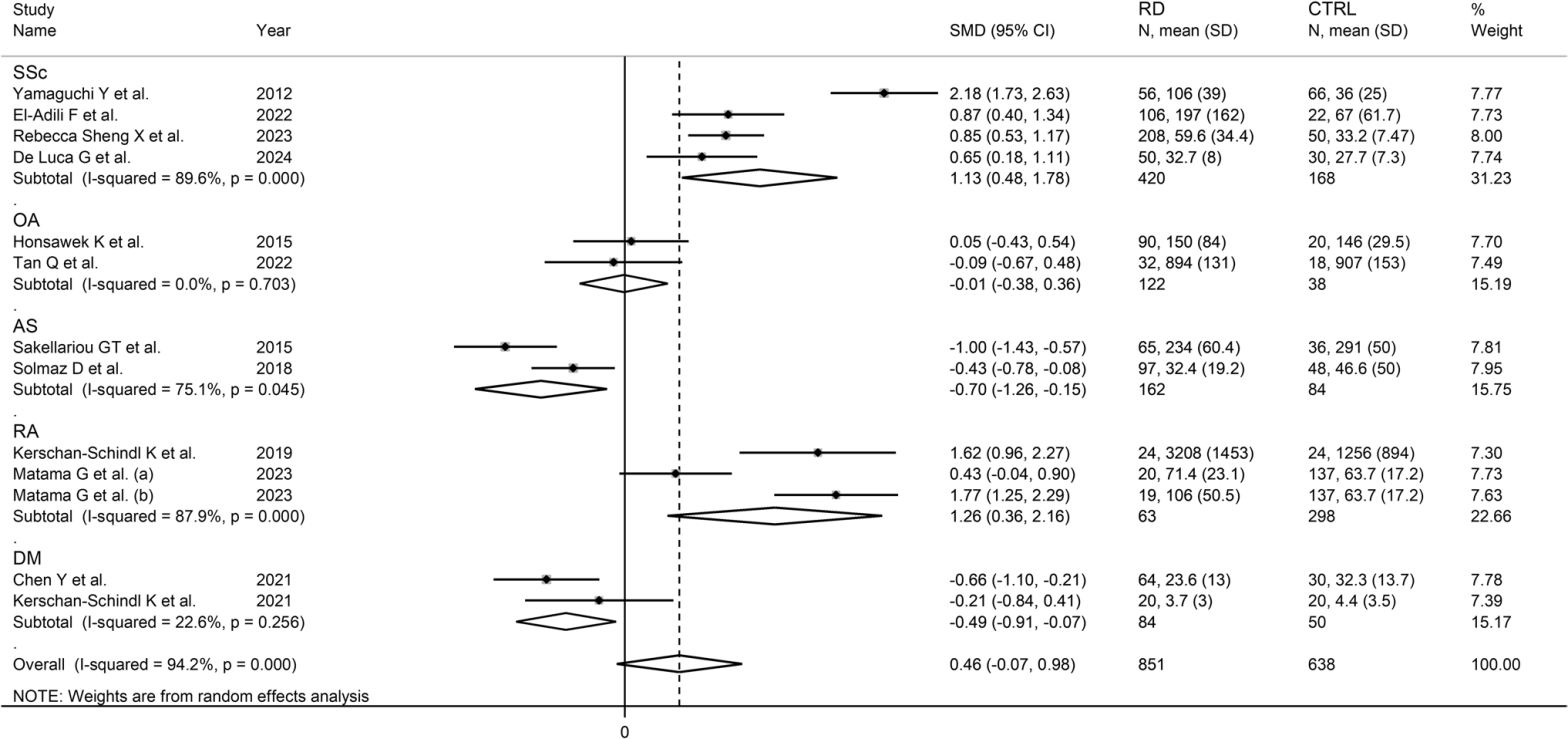
Forest plot of studies investigating periostin in patients with rheumatic diseases and healthy controls according to specific types of rheumatic disease

In univariate meta-regression, there were no significant associations between the effect size and age (*t* = 0.76, *p* = 0.47), male-to-female ratio (*t* = −1.70 *p* = 0.13), year of publication (*t* = 0.00, *p* = 1.00), or sample size (*t* = 0.74, *p* = 0.48). However, in subgroup analysis, the pooled SMD was significant in studies conducted in America (SMD = 0.85, 95% CI 0.59 to 1.12, *p* < 0.001; *I*^2^ = 0.00%, *p* = 0.95) but not Asia (SMD = 0.46, 95% CI −0.37 to 1.29, *p* = 0.27; *I*^2^ = 95.6%, *p* < 0.001) or Europe (SMD = 0.25, 95% CI −0.85 to 1.35, *p* = 0.66; *I*^2^ = 94.2%, *p* < 0.001), with a virtually absent heterogeneity in the American subgroup (Fig. 5). Moreover, significant RD-associated elevations in periostin concentrations were observed in studies of patients with systemic sclerosis (SMD = 1.13, 95% CI 0.48 to 1.78, *p* = 0.001; *I*^2^ = 89.6%, *p* < 0.001) and rheumatoid arthritis (SMD = 1.26, 95% CI 0.36 to 2.16, *p* = 0.006; *I*^2^ = 87.9%, *p* < 0.001) whereas no significant between-group differences were observed in studies of patients with osteoarthritis (SMD = −0.01, 95% CI −0.38 to 0.36, *p* = 0.96; *I*^2^ = 0.0%, *p* = 0.70), with a virtually absent heterogeneity observed in the last subgroup. Significant RD-associated reductions in periostin concentrations were observed in studies of patients with ankylosing spondylitis (SMD = −0.70, 95% CI −1.26 to −0.15, *p* = 0.013; *I*^2^ = 75.1%, *p* = 0.045) and dermatomyositis (SMD = −0.49, 95% CI -0.91 to -0.07, *p* = 0.022; *I*^2^ = 22.6%, *p* = 0.26), with a lower heterogeneity observed in the latter subgroup (Fig. 6).

### Certainty of evidence

The overall level of the certainty of evidence remained low (level 2) after considering the low-moderate risk of bias in all studies (no change), the high but partially explainable heterogeneity (no change), the lack of indirectness (no change), the moderate effect size (SMD = 0.46; no change) [44], and the absence of publication bias (no change).

## Discussion

This systematic review and meta-analysis provides early insights into the pathophysiological role of the matricellular protein periostin, which regulates inflammation, angiogenesis, and fibrosis, in RDs. Overall, a non-significant trend towards higher circulating periostin concentrations was observed in RD patients compared to controls. However, subgroup analysis revealed significant associations with specific types of RDs. Significant elevations in periostin concentrations were observed in studies of patients with rheumatoid arthritis and systemic sclerosis. By contrast, patients with osteoarthritis, ankylosing spondylitis, and dermatomyositis exhibited either similar or lower concentrations vs. controls. Further associations were observed with the geographical area where the research was conducted. There were significant RD-associated periostin elevations in American, but not Asian or European studies. Sensitivity analyses confirmed the stability of the results of the meta-analysis.

The expression of periostin is stimulated in pathological states by a wide range of factors, including interleukin 4 and 13, transforming growth factor beta, angiotensin 2, bone morphogenic protein 2, connective tissue growth factor 2, mechanical stretch, and several cancer-associated mediators [28]. The primary cell types involved in the synthesis of periostin are the fibroblasts, endothelial cells, and epithelial cells [31]. In turn, periostin can upregulate several cellular pathways, e.g. transforming growth factor beta, with the consequent shift from anti-fibrotic to pro-fibrotic states and the promotion of angiogenesis [64, 65], the PI3K/Akt pathway, which regulates cell proliferation, migration, survival, inflammation, and metabolism, and stimulates fibrosis [66, 67], the NF-κB pathway, a key regulator of immune and inflammatory responses, tumorigenesis, and fibrosis [68–70], and the Wnt and MAPK pathways, which also regulate cell proliferation, migration, apoptosis, and inflammation [71–74]. Taken together, the available evidence supports the complex yet influential role of periostin in regulating several pathophysiological processes favouring the onset and progression of several RDs.

The results of our subgroup analyses suggest that periostin is worth of further investigation as a potential biomarker of specific RDs, i.e. rheumatoid arthritis and systemic sclerosis. In addition to the intuitive role of periostin in favouring excess local and systemic inflammation, both conditions are also characterized by dysregulated angiogenesis and a pro-fibrotic tendency. Angiogenesis plays a critical role for the expansion and the creation of a pro-inflammatory state in the synovial tissue in patients with rheumatoid arthritis. These effects are primarily mediated by the VEGF and the fibroblast growth factor [75]. Furthermore, in addition to the well-known role of inflammation and fibrosis [76], dysregulated angiogenesis is also a key component of the pathophysiology of systemic sclerosis through excess synthesis of VEGF [77]. The pathophysiological role of VEGF in rheumatoid arthritis and systemic sclerosis is further supported by several systematic reviews and meta-analyses. The first reported significant circulating VEGF elevations in patients with rheumatoid arthritis (SMD = 1.48, 95% CI 0.82 to 2.15, *p* < 0.0001) and systemic sclerosis (SMD = 0.56, 95% CI 0.36 to 0.75, *p* < 0.0001) when compared to healthy subjects [78]. The second showed a significant and positive correlation between circulating VEGF concentrations and disease activity in rheumatoid arthritis (correlation coefficient = 0.66, 95% CI 0.281 to 0.446, *p* < 0.0001) [79]. The third reported significant VEGF elevations in patients with systemic sclerosis when compared to healthy controls (SMD = 0.93, 95% CI 0.71 to 1.15). In further analyses, VEGF concentrations were significantly higher in patients with diffuse disease compared to those with localized disease (SMD = 0.30, 95% CI 0.01 to 0.59, *p* = 0.046), in patients with late compared to active video capillaroscopy pattern (SMD = 0.35, 95% CI 0.09 to 0.61, *p* = 0.008), and in patients with pulmonary hypertension compared to those without (SMD = 0.93, 95% CI 0.34 to 1.53, *p* = 0.002) [80]. Notably, periostin has been shown to increase VEGF expression in cancer and other disease states [81, 82]. Pending additional studies, these observations support the proposition that the upregulation of periostin can directly or indirectly favour dysregulated inflammation, angiogenesis, and fibrosis, commonly observed in rheumatoid arthritis and systemic sclerosis. This hypothesis is further supported by the results of studies reporting the attenuation of experimental fibrosis and angiogenesis following periostin downregulation [83, 84]. By contrast, the lack of significant elevations or even a reduction in periostin concentrations in osteoarthritis, ankylosing spondylitis, and dermatomyositis, might reflect, in the presence of excess inflammation, a relatively lower pro-angiogenic and/or pro-fibrotic drive when compared to rheumatoid arthritis and systemic sclerosis [85–90]. Additional studies are warranted to test this hypothesis and also to investigate the concentrations of periostin in other RD types with different autoinflammatory, autoimmune, angiogenic, and fibrotic features. Such studies should provide useful information regarding whether periostin can facilitate diagnosis, predict clinical outcomes, and reflect treatment response in different types of RDs. The significant elevations in periostin observed in rheumatoid arthritis and systemic sclerosis in our systematic review and meta-analysis suggest its potential utility in diagnosing these RD types, as part of a comprehensive clinical and laboratory assessment. The further elevations observed in patients with rheumatoid arthritis with active disease [79] and patients with systemic sclerosis and diffuse disease and specific complications [80] also highlight the potential role of this matricellular protein in predicting clinical outcomes associated with these characteristics [91, 92]. Finally, the repeated measurement of periostin concentrations may allow determining specific disease trajectories and/or responses to different anti-inflammatory and immunomodulatory treatments.

Another interesting result in subgroup analysis was the association between the magnitude of the between-group differences in periostin concentrations and geographical location, with greater differences in studies conducted in America compared to those conducted in Asia and Europe. Epidemiological studies in patients with and without asthma have reported significantly higher periostin concentrations in non-asthmatic Chinese individuals when compared to non-asthmatic Caucasian participants [93]. Another study in patients with obstructive airways disease reported non-significant elevations in periostin concentrations in Asian participants when compared to European participants [94]. No significant differences in periostin concentrations between European, Māori, Pacific, and Asian participants were reported in another study in a cohort without asthma or chronic obstructive pulmonary disease [95]. More research is needed to confirm these findings in RD participants, including those from North and South America and other continents, e.g. Africa.

Our study has several strengths, including the assessment of periostin in different types of RDs, the investigation of associations between the effect size of between-group differences and several study and patient characteristics, and a rigorous evaluation of the risk of bias and the certainty of evidence. Important limitations include the relatively small number of studies, and the consequent restricted range of RDs selected for analysis, and the lack of evidence from specific geographical locations, e.g. Africa. For these reasons, the results of this systematic review and meta-analysis offer early, yet valuable, insights into the association between periostin and RDs which nevertheless warrants confirmation in further studies. Furthermore, the high heterogeneity observed highlights the variability in RD type and geographical settings. While subgroup analyses and meta-regressions provided insights into potential sources of variability, the interpretation of the pooled results requires caution. Future studies should aim to minimize heterogeneity by standardizing study protocols and investigating potential confounding factors in periostin measurement.

In conclusion, our study has shown the presence of significant elevations in circulating periostin, a protein modulating inflammation, angiogenesis, and fibrosis, in specific types of RDs, i.e. rheumatoid arthritis and systemic sclerosis. Additional research is required to confirm these observations and investigate the diagnostic and predictive role of periostin in a wider range of RDs and the potential influence of geographical factors and ethnicity. The results of such studies will be instrumental for determining whether periostin can serve as a candidate biomarker in specific RDs.

## Supplementary Information

Below is the link to the electronic supplementary material.

Supplementary file1 (DOCX 35 KB)

## Data Availability

The data supporting the findings of this systematic review and meta-analysis are available from AZ upon reasonable request.
